# Psychiatric comorbidity as predictor of costs in back pain patients undergoing disc surgery: a longitudinal observational study

**DOI:** 10.1186/1471-2474-13-165

**Published:** 2012-09-03

**Authors:** Alexander Konnopka, Margrit Löbner, Melanie Luppa, Dirk Heider, Sven Heinrich, Steffi Riedel-Heller, Hans Jörg Meisel, Lutz Günther, Jürgen Meixensberger, Hans-Helmut König

**Affiliations:** 1 Department of Medical Sociology and Health Economics, University Medical Centre Hamburg-Eppendorf, Hamburg, Germany; 2 Institute of Social Medicine, Occupational Health and Public Health, University of Leipzig, Leipzig, Germany; 3 Department of Neurosurgery, Berufsgenossenschaftliche Kliniken Bergmannstrost, Halle (Saale), Germany; 4 Department of Neurosurgery, Hospital St. Georg gGmbH, Leipzig, Germany; 5 Department of Neurosurgery, University of Leipzig, Leipzig, Germany

**Keywords:** Back pain, Disc surgery, Psychiatric comorbidity, Cost, Economic

## Abstract

**Background:**

Psychiatric comorbidity is common in back pain patients undergoing disc surgery and increases economic costs in many areas of health. The objective of this study was to analyse psychiatric comorbidity as predictor of direct and indirect costs in back pain patients undergoing disc surgery in a longitudinal study design.

**Methods:**

A sample of 531 back pain patients was interviewed after an initial disc surgery (T0), 3 months (T1) and 15 months (T2) using the Composite International Diagnostic Interview to assess psychiatric comorbidity and a modified version of the Client Sociodemographic and Service Receipt Inventory to assess resource utilization and lost productivity for a 3-month period prior interview. Health care utilization was monetarily valued by unit costs and productivity by labour costs. Costs were analysed using random coefficient models and bootstrap techniques.

**Results:**

Psychiatric comorbidity was associated with significantly (p < 0.05) increased direct (+664 Euro) and indirect costs (+808 Euro) at T0. The direct cost difference predominantly resulted from medical health care utilization and was nearly unchanged at T2. Further important cost predictors were clinical variables like the presence of chronic medical disease, the number of previous disc surgeries, and time and gender.

**Conclusion:**

Psychiatric comorbidity presents an important predictor of direct and indirect costs in back pain patients undergoing disc surgery, even if patients do not utilize mental health care. This effect seems to be stable over time. More attention should be given to psychiatric comorbidity and cost-effective treatments should be applied to treat psychiatric comorbidity in back pain patients undergoing disc surgery to reduce health care utilization and costs associated with psychiatric comorbidity.

## Background

In industrialized societies, back pain presents a common health problem, which is often associated with disc herniation [1]. While most back pain patients with disc herniation profit from a conservative treatment surgical interventions are performed in the most severe cases, when pain and sensory or motor deficits persist under conservative treatment [2]. It is well known that back pain and back pain treatment outcome are strongly influenced by psychological factors including psychiatric comorbidity [3-5]. Common psychiatric comorbidities in back pain patients with prevalence rates exceeding prevalence rates in the general population are affective disorders, anxiety disorders and substance abuse/dependency disorders [6,7]. Not only treatment outcome is associated with psychiatric comorbidity, but also health care costs. Several studies have found associations between psychiatric comorbidity and increased health care costs for different diseases, like dementia, substance abuse, heart failure or internal diseases [8-11]. In a recent study, Holmberg and Thelin [12] found that psychiatric comorbidity is associated with increased health care utilization in back pain patients. However, so far no study has analysed the effect of psychiatric comorbidity on health care costs in patients undergoing disc surgery in a prospective study design.

Estimating the costs of an illness is useful to inform decision makers about the economic relevance of a health problem and feasible objectives for interventions. Additionally, cost-of-illness studies provide input data for further research, in particular for studies modelling cost-effectiveness of interventions. In general, costs of illness can be distinguished into direct costs and indirect costs. Whereas direct costs refer to the monetary value of utilized resources (e.g. for hospital stays, physician visits or drugs), indirect costs refer to productivity lost due to morbidity or premature mortality.

The aim of our study was to analyse predictors for direct and indirect costs in back pain patients undergoing disc surgery, and in particular the effect of psychiatric comorbidity in a prospective study design. Thereby, we hypothesised that psychiatric comorbidity is connected to increased direct as well as indirect costs.

## Methods

### Study design and sample

Our study referred to 620 consecutive back pain patients who underwent an index treatment of surgery for herniated disc between April 2007 and October 2008. The study received ethics committee approval by the ethics committee of the University of Leipzig. We included patients who provided informed consent, were between the age of 18 and 55 years, spoke German sufficiently and had a radiological determined herniated disc of the lumbal or cervical spine. Study participation was declined by 86 (14%) of the 620 patients and 3 patients were excluded due to missing data on psychiatric comorbidity, resulting in a final sample of 531 patients. Disc surgery was either conducted at the Department of Neurosurgery at the University Hospital of Leipzig (N = 149, 28%), the Department of Neurosurgery at the Hospital St. Georg, Leipzig (N = 153, 29%) or the Department of Neurosurgery at the Hospital “Bergmannstrost”, Halle (N = 229, 43%). After disc surgery, all patients were offered a rehabilitation to prepare return to work which was rejected by 33 patients (7%) of 485 non-drop-out patients available at T1. Included patients were interviewed face-to-face by experienced and trained psychologists about psychiatric comorbidity and health care utilization approximately 3.63 (SD 2.83) days after disc surgery (T0). Follow up interviews were conducted 3 months (T1) and 15 months (T2) post treatment via telephone.

### Instruments

#### Socio-demographic and illness-related variables

As sociodemographic variables, age, gender, education, living situation, marital and employment status were assessed. Patients were classified according to the type of disc herniation (cervical or lumbar) and were asked about the number of previous disc herniations as well as previous disc surgeries in their case history. Furthermore, presence and nature of other chronic medical diseases were assessed.

#### Psychiatric comorbidity

We assessed psychiatric comorbidity with a German version of the Composite International Diagnostic Interview (CIDI) [13]. The CIDI is a standardized, fully structured diagnostic interview for the assessment of mental disorders that provides current and lifetime diagnoses according to the definitions of ICD-10 and DSM-IV. For the purpose of our study retrospective 6-month prevalence data were assessed at baseline. We assessed the CIDI sections for affective, anxiety and substance related disorders as the most common comorbid conditions in backpain patients, as described by Härter et al. [14]. Patients were asked probe questions on core symptoms of these psychiatric disorder groups. If such a symptom required medical attention or was sufficiently severe to affect daily life, it was scored as present, and a more detailed investigation was conducted. Finally, information on the onset and the recent nature of the particular cluster of symptoms were assessed to generate prevalence data.

#### Resource utilization and loss of productivity

At every measurement, resource utilization and loss of productivity were retrospectively measured for the preceding three months using a standardized questionnaire that was adapted from questionnaires used in earlier studies [15-23]. Hence T0 covered a 3 month period prior the surgical intervention, T1 covered the 3 months following the surgical intervention and T2 covered a 3 month period starting 1 year after the surgical intervention. We assessed utilization of inpatient treatment, rehabilitation, outpatient physician and non-physician services, medical goods and dentures, drugs, transports to medical treatment and informal care by relatives and/or friends. We further assessed loss of productivity due to sickness absence days, early retirement, time spent for treatments and productivity reduction at work. Patients were asked to indicate their disease related productivity reduction at work on a scale ranging from “no reduction of productivity” to “unable to work”.

### Cost calculation

Costs were calculated from a societal perspective by valuing resource utilization by corresponding unit costs and productivity losses by age and gender specific labour costs. For index treatment individual hospital billing information were available and used to calculate rates for diagnoses related groups (DRG) [24,25]. Other hospital treatments and the post-surgery rehabilitation were valued by hospital-type specific mean rates per diem [26,27]. Outpatient physician and non-physician visits were valued by type specific costs per contact [26,28]. Drug costs were calculated using prices from the German catalogue of drugs “red list” [29]. For drugs not listed in the red list, as well as medical goods and dentures we used market prices. Informal care was valued by average German net earnings [26] representing the opportunity costs of leisure time. Costs for transportation were asked from the patients, except for car use which was valued by 0.30 Euro per kilometre according to the German income tax act [30]. Lost productivity was valued by age and gender specific gross earnings [31] plus additional employer payments for social insurances [32]. Except for medical goods, dentures and pharmaceuticals not listed in the red list, all costs refer to the year 2007 (year of measurement). For medical goods, dentures and pharmaceuticals not listed in the red list, prices were obtained from an internet based research for actual market prices. These prices refer to 2008 and 2009, corresponding to the specific time point of the searches. Unit costs originally estimated before 2007 were inflated to 2007 values using the German consumer price index [33] for direct costs and the growth rate of earnings in Germany [34] for indirect costs.

### Lost to follow up analyses

From the 531 patients included at T0, 485 (91%) were available for interviews at T1 and 286 at T2 (54%). All patients lost at T1 and 88 of the patients lost at T2 were drop outs, whereas 157 of the patients lost at T2 were not interviewed because of study end. There were no significant differences between complete follow up participants (N = 286) and patients lost to follow up (N = 245) regarding disc location, gender, age, psychiatric comorbidity, presence of chronic medical disease, the number of previous disc herniations or surgeries, number of children, education, marital status, living status or employment status at baseline. The only significant (p < .05) difference was for type of health care insurance with patients lost to follow up being more often members of the statutory health insurance.

### Missing values

To be able to calculate overall direct and indirect costs for a sufficient sample size, we were forced to replace missing values for single variables. Table 1 shows maximum percentages of missing values for single cost categories. For most variables a maximum of three percent of values was missed, except for the DRG rates of index treatment (21% missings) and the number of drug packages used (9.6% missings at T1, 7.0% missings at T2). Missed DRG rates were replaced by the diagnosis-specific (lumbal or cervical) mean. Due to the very large heterogeneity of drugs consumed, we decided to stay conservative and replaced missing numbers of drug packages by a value of one. For all other variables, missing values of an indicated utilization were replaced by the mean utilization of respective users. If, for example, a patient indicated outpatient physician visits but not the number of visits, we replaced this missing value by the average number of visits of physician visitors.

**Table 1:** Missing values of resource utilization

	**T0**		**T1**		**T2**	
	**N**	**Max % missings**	**N**	**Max % missings**	**N**	**Max % missings**
Hospital fee data for DRG calculation of index treatment	531	21.09	-	-	-	-
Other hospital related variables	531	0.19	484	0.41	284	0
Rehabilitation related variables	-	-	484	0.21	284	0
Outpatient physician visits	531	0	484	0.41	284	0.70
Outpatient non-physician visits	531	0.19	484	0.62	284	1.06
Number of drug units consumed	1,455^a^	2.27	635^a^	9.61	588^a^	6.97
Medical goods consumed	531	0	484	0	284	1.06
Informal care related variables	531	0.75	484	2.69	284	0.35
Transports related variables	531	0.56	484	2.48	284	1.76
Loss of productivity related variables	427^b^	2.34	357^b^	2.80	203^b^	2.46

^a^ number and percentage refer to the overall number of drug labels reported; ^b^ percentage refers to the number of employed.

### Statistical analysis

Differences in sociodemographic variables between patients with/without psychiatric comorbidity were analysed via *t*-test and chi-square test as appropriate. We used random coefficient models to test our hypothesis and to identify explanatory variables of direct, indirect and total costs. Since cost data are often characterized by non-normality and right-skewness, we used bootstrap techniques (4,000 replications) to estimate standard errors [35]. Explanatory variables used were type of disc herniation, psychiatric comorbidity, time of measurement, the interaction effect of psychiatric comorbidity and time, presence of other chronic medical diseases, the number of previous disc surgeries, age, gender, education, marital status, living situation, employment status, type of health insurance (statutory health insurance or private health insurance) and the number of children. In a base case scenario, we analysed direct costs without informal care (due to the hypothetic nature of informal care costs) and indirect costs without reduction of productivity in work (due to limited validity of our assessment method). Analyses including informal care costs and reduced productivity were conducted as alternative scenario. Significance level was set at p < 0.05. Cost were calculated using PASW (PASW Statistics 18), statistical analyses were performed using STATA (STATA, Release 10.0).

## Results

### Sample characteristics

Sample characteristics are shown in Table 2. Overall, mean age was 42.4 years, 57% of patients were male, 16.2% had psychiatric comorbidity and 38.8% had comorbid chronic medical diseases. On average, every fifth patient had received a former disc surgery in the past.

**Table 2:** Sample characteristics at T0

	**Overall (N = 531)**			**Lumbal disc (N = 419)**			**Cervical disc (N = 112)**		
	**Without psychiatric comorbiditiy (N = 445)**	**With psychiatric comorbidity (N = 86)**		**Without psychiatric comorbiditiy (N = 352)**	**With psychiatric comorbidity (N = 67)**		**Without psychiatric comorbiditiy (N = 93)**	**With psychiatric comorbidity (N = 19)**	
Age (mean (SD))	42.6 (7.87)	41.4 (8.34)	^a^	41.8 (8.05)	40.5 (8.78)	^a^	45.5 (6.35)	44.6 (5.63)	^a^
Number of children (mean (SD)	1.41 (0.98)	1.43 (2.28)	^a^	1.37 (1.00)	1.13 (0.99)	^a^	1.55 (0.89)	2.47 (4.41)	^a^
Number of previous disc herniations (mean (SD))	1.61 (1.62)	2.70 (10.3)	^a^	1.64 (1.73)	3.00 (11.6)	^a^^*^	1.51 (1.12)	1.59 (1.33)	^a^
Number of previous disc surgery (mean (SD))	0.22 (0.58)	0.29 (0.62)	^a^	0.22 (0.58)	0.37 (0.68)	^a^	0.18 (0.57)	0 (0)	^a^^**^
Psychiatric comorbidity									
---ICD-10 chapter F0 (N (%))	-	3 (1)		-	3 (1)		-	0 (0)	
---ICD-10 chapter F1 (N (%))	-	11 (2)		-	7 (2)		-	4 (4)	
---ICD-10 chapter F3 (N (%))	-	56 (11)		-	44 (11)		-	12 (11)	
---ICD-10 chapter F4 (N (%))	-	47 (9)		-	38 (9)		-	9 (8)	
Self reported chronic medical disease (N (%))									
---Yes (ref: No)	170 (38)	36 (42)	^b^	229 (65)	45 (67)	^b^	46 (49)	5 (26)	^b^
Gender (N (%)									
---Male	179 (60)	47 (45)	^b^*	^b^^*^ 214 (61)	32 (48)	^b^^*^	52 (56)	7 (37)	^b^
Education (N (%))									
---ISCED level 2 or lower	7 (2)	4 (5)	^b^	6 (2)	4 (6)	^b^	1 (1)	0 (0)	^b^
---ISCED level 3	306 (69)	57 (66)		240 (68)	48 (72)		66 (71)	9 (47)	
---ISCED level 5 or higher	132 (30)	25 (29)		106 (30)	15 (22)		26 (28)	10 (53)	
Marital status (N (%))									
Married (ref: alone)	264 (59)	35 (41)	^b^**	^b^^**^205 (58)	23 (34)	^b^^***^	59 (63)	12 (63)	^b^
Living situation (N (%))									
---Living alone	68 (15)	24 (28)	^b^**	^b^^**^53 (15)	19 (28)	^b^^*^	15 (16)	5 (26)	^b^
---Living with partner/parents	364 (82)	58 (67)		290 (82)	45 (67)		74 (80)	13 (68)	
---Other living situation	13 (3)	4 (5)		9 (3)	3 (4)		4 (4)	1 (%)	
Employment status (N (%))									
---Employed (ref: unemployed)	371 (83)	54 (63)	^b^***	^b^^***^296 (84)	40 (59)	^b^^***^	75 (81)	14 (74)	^b^
Type of health insurance (N (%))									
---Statutory health insurance	381 (86)	81 (94)	^b^*	^b^^*^308 (87)	62 (93)	^b^	73 (78)	19 (100)	^b^^*^
---Private health insurance	64 (14)	5 (6)		44 (13)	5 (7)		20 22)	0 (0)	

^a^*T*-test; ^b^ Chi-square test; ISCED: international standard classification of education; SD: standard deviation; * p < .05; ** p < .01; *** p < .001.

The most prevelant comorbidity was depression (11%) followed by anxiety disorders (9%) and substance abuse disorders (2%). There were no relevant differences in comorbidity between patients with lumbal and patients with cervical disc herniation.

We found several statistically significant differences between patients with and without psychiatric comorbidity. Overall, patients with psychiatric comorbidity were more often female, single, living alone, were more often unemployed and members of statutory health insurance. Further, in lumbal disc herniation patients with psychiatric comorbidity had statistically significant more previous disc herniation than patients without psychiatric comorbidity.

### Costs

Overall, mean 3-month direct costs were 5,403 Euro (SD: 2,238 Euro) at T0, 2,862 Euro (SD: 1,932 Euro) at T1 and 811 Euro (SD: 2,191 Euro) at T2, whereas mean 3-month indirect costs were 4,130 Euro (SD: 4,083 Euro) at T0, 6,629 Euro (SD: 4,861 Euro) at T1 and 1,623 Euro (SD: 3,673 Euro) at T2.

The results of our base case regression analysis are shown in Table 3. Direct costs were strongly predicted by time and “clinical” variables (disc location, psychiatric comorbidity, chronic medical disease, number of previous disc surgeries). Time was the most prominent cost predictor due to changes in therapy over time (T1: -2,456 Euro; T2: -4,634 Euro). Psychiatric comorbidity at T0 was the second strongest predictor of direct costs (+664 Euro), even stronger than the presence of chronic medical disease (+467 Euro). Interaction effects of time and psychiatric comorbidity at T0 were not statistically significant for direct costs.

**Table 3:** Predictors of direct, indirect and total costs; base case scenario

**Parameter**	**Direct costs**	**Standard error**		**Indirect costs**	**Standard error**		**Total costs**	**Standard error**	
Constant	4,762	559	***	3,998	768	***	8,757	976	***
Diagnosis	478	128	***	6.52	231		509	280	
Psychiatric comorbidity	664	265	*	808	376	*	1,469	528	**
T1	−2,456	130	***	3,028	235	***	571	291	*
T1 x psychiatric comorbidity	−578	350		−1,248	600	*	−1,829	783	*
T2	−4,634	164	***	−2,370	288	***	−6,996	370	***
T2 x psychiatric comorbidity	72	517		1,333	833		1,355	1,089	
Chronic medical disease	467	155	**	951	249	***	1,417	317	***
Number of previous disc surgeries	503	141	***	1,142	167	***	1,645	228	***
Age (centralized to mean)	−6.28	7.66		48	14	***	41	17	*
Gender (ref: female)	−354	130	**	1,572	189	***	1,203	246	***
Being married	95	137		363	273		462	333	
ISCED educational level 3 (ref ≤ 2)	451	401		−280	634		132	762	
ISCED educational level 5 (ref ≤ 2)	367	414		−790	665		−460	803	
Living situation: with partner (ref: alone)	−325	231		87	359		−180	440	
Living situation: others (ref: alone)	−52	458		344	919		374	1,060	
Number of children	64	59		−249	102	*	−196	128	
Private health insurance (ref: statutory health insurance)	−57	139		−759	279	**	−799	340	*
Being not employed (ref: being employed)	238	196		−4,502	331	***	−4,228	409	***
Model statistics									
R2 within	0.55			0.42			0.46		
R2 between	0.33			0.28			0.26		
R2 overall	0.46			0.34			0.36		

* p < .05; ** p < .01; *** p < .001.

Also with indirect costs, clinical variables like psychiatric comorbidity at T0 (+808 Euro), chronic medical disease (+951 Euro) and the number of previous disc surgeries (+1,142 Euro) were important cost predictors. However, the most important cost predictors were employment status (−4,502 Euro if unemployed) and time (T1: +3,028 Euro; T2: -2,370 Euro). At T1, the interaction of time and psychiatric comorbidity at T0 reached significance level for indirect costs. Here psychiatric comorbidity at T0 was associated with a reduction in indirect costs (−1,248 Euro). Further significant predictors of indirect costs were the number of children (−249 Euro), private health insurance (−759 Euro) and gender (+1,572 Euro). For total costs psychiatric comorbidity at T0 (+1,469 euro), chronic medical disease (+1,417 Euro), the number of previous disc surgery (+1,645 Euro) and male gender (+1,203 Euro) were almost equally strong explanatory variables.

In the alternative scenario with informal care included in direct costs and reduction of productivity in work included in indirect costs, only few changes occurred (Table 4). There were no changes in signs of any statistical significant explanatory variable, but psychiatric comorbidity was not a significant predictor of indirect costs any more. For both direct as well as indirect costs, inclusion of the two additional cost categories resulted in a larger constant, and in partially larger effects in particular of employment status and gender.

**Table 4:** Predictors of direct, indirect and total costs including monetarily valued informal care and reduced productivity at work

**Parameter**	**Direct costs**	**Standard error**		**Indirect costs**	**Standard error**		**Total costs**	**Standard error**	
Constant	6,388	869	***	6,915	635	***	13,269	1,176	***
Diagnosis	381	158	*	−28	205		371	277	
Psychiatric comorbidity	893	352	*	448	327		1,348	549	*
T1	−2,450	166	***	994	201	***	−1,453	283	***
T1 x psychiatric comorbidity	−564	454		−688	499		−1,242	755	
T2	−5,063	192	***	−3,112	282	***	−8,167	368	***
T2 x psychiatric comorbidity	38	709		1,646	762	*	1,643	1,133	
Chronic medical disease	643	191	***	811	229	***	1,397	329	***
Number of previous disc surgeries	548	162	***	1,197	147	**	1,755	232	***
Age (centralized to mean)	−9.50	10.30		67	12.40	***	58	17.69	**
Gender (ref: female)	−871	158	***	2,237	167	***	1,343	247	***
Being married	104	181		499	262		594	354	
ISCED educational level 3 (ref ≤ 2)	−484	711		256	521		−209	972	
ISCED educational level 5 (ref ≤ 2)	−681	729		−160	550		−829	1,009	
Living situation: with partner (ref: alone)	−212	269		−100	337		−260	470	
Living situation: others (ref: alone)	580	646		689	929		1,333	1,233	
Number of children	87	79		−328	90	***	−235	130	
Private health insurance (ref: statutory health insurance)	−40	165		−740	255	**	−775	326	*
Being not employed (ref: being employed)	691	269	*	−7,025	323	***	−6,347	462	***
Model statistics									
R2 within	0.48			0.46			0.53		
R2 between	0.33			0.52			0.35		
R2 overall	0.41			0.48			0.43		

* p < .05; ** p < .01; *** p < .001.

## Discussion

The aim of this study was to investigate whether psychiatric comorbidity predicts direct and indirect cost in back pain patients undergoing disc surgery and to estimate other predictors for direct and indirect costs, in a prospective study design. Summarized, we found that direct as well as indirect costs were strongly predicted by time and clinical variables including psychiatric comorbidity.

We found that psychiatric comorbidity is associated with increased direct costs, though we found almost no mental health care utilization at all measurements, i.e. psychiatric comorbidity increased non-mental health care utilization, which has also been found in other diseases [8-11]. There are several possible explanations for this finding. One may be, that psychiatric comorbidity is often either not diagnosed or not treated with psychiatric services, as shown in other studies [36,37]. Another possible explanation may be underreporting of this specific type of health care due to fear of stigma. Finally, stigma may also be a reason why patients do not utilize psychiatric treatment.

As a result of the therapeutic pathway, time was a very strong predictor of costs. At T0 direct costs were strongly influenced by acute disease and in particular by disc surgery resulting in the highest three month health care costs of all measurements. Acute disease and disc surgery were both also associated with sickness absence causing high indirect costs at T0. At T1 direct costs primarily resulted from inpatient rehabilitation and subsequent outpatient treatments, resulting in considerably lower direct costs compared to T0. Indirect costs strongly increased at T1 due to more sickness absence days resulting from postoperative sick leave and inpatient rehabilitation which lasted several weeks. At T2 acute treatment and rehabilitation had been completed and direct costs preliminary consisted of outpatient treatment costs, resulting in the lowest three month health care costs of all periods analysed. Accordingly, most patients were back to work again and sickness absence (and indirect costs) declined considerably.

Though mostly not significant, the interaction term of time and psychiatric comorbidity showed an identical interesting course over time for direct and indirect costs: at T0 patients with psychiatric comorbidity showed higher costs than patients without; at T1 this difference diminished, but was found again at T2. In our opinion, this course could be seen as an artefact resulting from the rather strictly organised therapy flow during the 3-month interval preceding the T1 assessment. This interval was characterized by the postoperative therapy and an inpatient rehabilitation lasting mostly three to four weeks, which both are highly standardized. On the one hand this limited patient’s choice of health care utilization and thus equalized direct costs of patients with and without psychiatric comorbidity. On the other hand, relative standardized durations of postoperative sick leave and rehabilitation, may also have equalized indirect costs of patients with and without psychiatric comorbidity. However, at T2 this “effect” of therapy was not present anymore; hence the impact of psychiatric comorbidity on direct as well as indirect costs was observable again. In conclusion the impact of psychiatric comorbidity on cost seems to have persisted after disc surgery and rehabilitation.

Besides time and psychiatric comorbidity, direct and indirect costs were also significantly associated with the number of previous disc surgeries: the more disc surgeries patients received in the past, the higher costs occurred. Having multiple disc surgeries indicates a worse health state, e.g. due to a chronic back pain disease with more severe spine involvement or more complicated surgery conditions. This may result in higher direct costs due to more treatments and higher indirect costs due to more sickness absence.

Our regression analysis showed significant associations of direct and indirect costs with gender. Female gender was associated with higher direct but lower indirect costs. A deeper view into direct costs showed that women had higher costs in almost all cost categories for lumbal and cervical disc herniations regardless of psychiatric comorbidity being present or not. One possible explanation for this finding may be that women in our sample were in worse health states. Women had more often comorbid chronic medical conditions (44% vs. 35%) and received on average more previous disc surgeries (0.29 vs. 0.19). Both variables were associated with higher direct costs in the regression analysis which may partially explain the gender effect. For indirect costs, we interpret the finding of lower costs in women - at least in part - as an artefact resulting from lower productivities applied for the monetary valuation of lost productivity time.

Interestingly, indirect costs were significantly (p < 0.05) negatively associated with the number of children and private health insurance. We interpret these two findings as results of selection bias. On the one hand, one could assume that patients with children have more pressure to return to work, which may result in reduced indirect costs. On the other hand members of private health insurance tend to be healthier due to risk selection of private insurers. Further, members of private health insurance in Germany often earn higher income or are self-employed which both may be incentives to return to work fast.

In our base case analysis we excluded direct informal care costs and indirect costs resulting from reduced productivity at work. Costs of informal care were excluded because they are somewhat hypothetic: informal care costs present monetarily valued care time of relatives or friends [38]. Thus – in contrast to all other direct costs – no “real” money is paid. Instead, informal care costs represent the opportunity costs of leisure time lost by relatives or friends. Productivity reduction at work was assessed by a ten step Likert scale ranging from “no reduction of productivity” to “unable to work” on which patients were asked to rate themselves. This scale has not been validated yet, therefore we excluded productivity reduction at work from our base case analysis too. Including these two cost categories in alternative analyses primarily resulted in clearly larger constants and larger effects of employment status and gender but no fundamental differences in results like sign changes.

Our statistical models explained a great share of the overall variance in costs, with coefficients of delimination (R^2^) ranging from 0.34 to 0.48. One must note that these high values of R^2^ are in part a result of pseudo-variance generated by the variables disc location and employment status. Whereas average costs of disc surgery were 3,572 Euro for lumbal disc herniation, they were 5,618 Euro for cervical disc herniation. Indirect costs predominantly occurred in employed patients resulting in average 3-month indirect costs of 5,338 Euro for those employed compared to 1,907 Euro for those unemployed. Thus the relative high values of R^2^ generated by our models should be seen with caution.

Our study has some limitations. We found relative high portions of missing values in DRG rates and the number of drug packages used. DRG coding required a complete set of variables, including hospital record data which were often not available, whereas the high portion of missings for drug packages may be due to memory effects. Our sample contained patients with cervical and lumbal disc herniations, which may bias our results. However, disease specific and sociodemographic characteristics were similar in both patient groups; furthermore we controlled for disc location in our regression analysis. Our assessment of psychiatric comorbidity was restricted to the most important CIDI sections (affective, anxiety and substance use disorders) that represent the most prevalent and costly psychiatric disorders. Further, the assessment of psychiatric comorbidity took place after the surgical intervention and may be influenced by this acute event, resulting in an overestimation of prevalence rates. Finally, some prices were not from our base year 2007, because no prices for this year were available. Instead we were forced to use prices of 2008 and 2009 for some goods. However, the portion of costs affected by this bias was very low und should not have a significant effect on the results.

### Implications for clinical practice

Our findings imply that more attention should be given to psychiatric comorbidity in the back pain patients undergoing disc surgery. Clinicians should be aware of the high prevalence rates of psychiatric comorbidity in back pain patients, in particular in the most severe cases which are treated via surgery. If applicable, they should consider the assessment of psychiatric distress and support of mental health professionals [39]. Multimodal diagnostic and therapy approaches that pay attention to psychiatric comorbidity may help to improve the outcomes of surgical therapy and to reduce the costs connected to psychiatric comorbidity.

## Conclusion

We found a strong effect of psychiatric comorbidity on direct as well as indirect costs in back pain patients undergoing disc surgery. Yet utilization of psychiatric treatments was negligible. The cost effect of psychiatric comorbidity decreased during the rehabilitation period but was present again at follow up. More attention should be given to psychiatric comorbidity in disc surgery patients and mental health care services should be offered to these patients.

## Competing interest

The authors declare that they have no competing interests.

## Authors’ contributions

AK calculated and analysed the cost data, participated in the interpretation of results and prepared the manuscript. MZ and ML prepared clinical questionnaires, collected data and participated in the interpretation of results. DH was the primary statistician and conducted all final statistical analyses. SH contributed to the health economic study design and designed the health economic questionnaire. HJM, LG and JM contributed to the study design and recruited study patients. SRH contributed to the clinical study design and management, and contributed to the interpretation of results. HHK contributed to the health economic study design and management, and interpretation of results. All authors read and approved the final manuscript.

## Pre-publication history

The pre-publication history for this paper can be accessed here:

http://www.biomedcentral.com/1471-2474/13/165/prepub
